# Using hypnosis to disrupt face processing: mirrored-self misidentification delusion and different visual media

**DOI:** 10.3389/fnhum.2014.00361

**Published:** 2014-06-18

**Authors:** Michael H. Connors, Amanda J. Barnier, Max Coltheart, Robyn Langdon, Rochelle E. Cox, Davide Rivolta, Peter W. Halligan

**Affiliations:** ^1^ARC Centre of Excellence in Cognition and its DisordersSydney, NSW, Australia; ^2^Department of Cognitive Science, Macquarie UniversitySydney, NSW, Australia; ^3^Dementia Collaborative Research Centre, School of Psychiatry, University of New South WalesSydney, NSW, Australia; ^4^School of Psychology, University of East LondonLondon, UK; ^5^Department of Neurophysiology, Max Planck Institute for Brain ResearchFrankfurt am Main, Germany; ^6^Ernst Strüngmann Institute for Neuroscience in Cooperation with Max Planck SocietyFrankfurt am Main, Germany; ^7^School of Psychology, Cardiff UniversityCardiff, UK

**Keywords:** delusion, face perception, hypnosis, instrumental hypnosis, mirror sign, mirrored-self misidentification, self-recognition, visual self-recognition

## Abstract

Mirrored-self misidentification delusion is the belief that one’s reflection in the mirror is not oneself. This experiment used hypnotic suggestion to impair normal face processing in healthy participants and recreate key aspects of the delusion in the laboratory. From a pool of 439 participants, 22 high hypnotisable participants (“highs”) and 20 low hypnotisable participants were selected on the basis of their extreme scores on two separately administered measures of hypnotisability. These participants received a hypnotic induction and a suggestion for either impaired (i) self-face recognition or (ii) impaired recognition of all faces. Participants were tested on their ability to recognize themselves in a mirror and other visual media – including a photograph, live video, and handheld mirror – and their ability to recognize other people, including the experimenter and famous faces. Both suggestions produced impaired self-face recognition and recreated key aspects of the delusion in highs. However, only the suggestion for impaired other-face recognition disrupted recognition of other faces, albeit in a minority of highs. The findings confirm that hypnotic suggestion can disrupt face processing and recreate features of mirrored-self misidentification. The variability seen in participants’ responses also corresponds to the heterogeneity seen in clinical patients. An important direction for future research will be to examine sources of this variability within both clinical patients and the hypnotic model.

## INTRODUCTION

Hypnotic suggestions can temporarily disrupt or alter many cognitive processes (Hilgard, 1965; Kihlstrom, 1985, 2007; Oakley and Halligan, 2009, 2013). In visual perception, for example, specific hypnotic suggestions can cause participants to hallucinate (Szechtman et al., 1998), become blind (Bryant and McConkey, 1999), or selectively ignore particular areas of their visual field (Oakley and Halligan, 2009; Priftis et al., 2011). These experiences can be very compelling – to the point that many participants have difficulty distinguishing the hypnotically suggested alterations from reality (Woody and Szechtman, 2000, 2011; Bryant and Mallard, 2003) – yet are completely reversible (Hilgard, 1965; Kihlstrom, 1985, 2007). In some cases, these alterations may even reflect changes to otherwise automatic cognitive processes (Lifshitz et al., 2013). Hypnotic suggestion is thus a powerful tool to manipulate and study cognition (Oakley and Halligan, 2009, 2013). One such application is in the study of clinical disorders (Kihlstrom, 1979). In previous work, we used hypnotic suggestion to disrupt self-recognition and “model” the neuropsychiatric mirrored-self misidentification delusion, the belief that one’s reflection in the mirror is a stranger (e.g., Connors et al., 2012a). The current experiment extends this work by using hypnotic suggestion to disrupt face processing while testing both self-recognition and face recognition across different visual media.

### MODELLING MIRRORED-SELF MISIDENTIFICATION DELUSION

Mirrored-self misidentification delusion commonly occurs in dementia. Approximately 2–7% of patients with Alzheimer’s disease misidentify their own reflection in the mirror (see Connors and Coltheart, 2011; Connors et al., in press-b). The delusion can also occur in schizophrenia (Gluckman, 1968) and after stroke (Villarejo et al., 2011). Patients vary in their reactions to the “stranger.” Some patients treat their reflection as a companion (Phillips et al., 1996). Other patients remain indifferent (Breen et al., 2001) or are deeply suspicious of the stranger (Gluckman, 1968). The delusion can occur despite intact semantic knowledge of mirrors (e.g., being able to define their properties and function; Breen et al., 2001). The delusion can also occur despite an ability to accurately recognize *other* people’s reflections in the mirror (Spangenberg et al., 1998; Breen et al., 2001; Villarejo et al., 2011).

The influential two-factor theory of clinical delusions provided by Langdon and Coltheart, 2000 (see also Coltheart et al., 2011) proposes that two separate factors are necessary for a delusion. The first factor (Factor 1) explains the *content* of a delusion and typically involves some type of perceptual and/or emotional anomaly. In the case of mirrored-self misidentification, either impaired face processing (which leads to a difficulty in recognizing one’s own face in the mirror) or mirror agnosia (an inability to use mirror knowledge when interacting with mirrors) can lead to the idea that there is a stranger in the mirror (Breen et al., 2001). The second factor (Factor 2) explains why the delusion is *maintained* and involves a deficit in belief evaluation. This second factor accounts for why some patients with impaired face processing or mirror agnosia develop a delusion and others do not (for a description of patients with these deficits without the delusion, see Ellis and Florence, 1990; Connors and Coltheart, 2011). The second factor may result from damage to the prefrontal cortex. This damage may be specific to the right dorsolateral prefrontal cortex (Coltheart, 2010), though it might also involve other areas, such as the ventromedial prefrontal cortex (Gilboa, 2010; Turner and Coltheart, 2010) or right inferior frontal gyrus (Sharot et al., 2011).

Delusions are difficult to study because of co-occurring symptoms and impairments. Mirrored-self misidentification delusion, in particular, is difficult to study because of the cognitive and neurological deterioration associated with dementia. Hypnotic suggestion allows researchers to recreate critical aspects of the delusion while avoiding some of these challenges (Kihlstrom, 1979; Kihlstrom and Hoyt, 1988; Cox and Barnier, 2010; Connors, 2012). Hypnotic suggestion is able to recreate many of the “surface features” of mirrored-self misidentification. The majority of high hypnotisable participants (“highs”), for example, who are hypnotized and given a suggestion to see a stranger in a mirror, report this experience and show features strikingly similar to clinical patients (Barnier et al., 2008, 2011). Participants, for example, maintain this belief when challenged and interact with their reflection as if it were another person.

Hypnosis may also be able to model the underlying neuropsychological processes of mirrored-self misidentification delusion as specified by the two-factor theory. Whereas a suggestion for impaired face processing or mirror agnosia may produce the content of the delusion (Factor 1), hypnosis by itself may disrupt belief evaluation (Factor 2; Connors et al., 2012a,b, 2013). People tend to accept ideas during hypnosis that they would normally reject in an ordinary, everyday state of consciousness (Shor, 1959). In support of this, previous research has shown that a hypnotic induction by itself reduces the ability of highs to distinguish between suggested and real events (Bryant and Mallard, 2003); encourages more holistic, rather than detail-oriented, processing of visual memory (Crawford and Allen, 1983); and affects brain areas, such as the upper pons, thalamus, rostral areas of the right anterior cingulate cortex, prefrontal cortex, and right inferior parietal lobule, that are involved in attention, absorption, and critical thinking (Rainville et al., 2002; Oakley, 2008; Deeley et al., 2012). Our previous research has compared participants given suggestions either with or without hypnosis to manipulate Factor 2 and demonstrated that hypnosis is necessary for most participants to experience the delusion (Connors et al., 2012a, 2013). Specific suggestions within hypnosis may thus allow researchers to create a laboratory model of mirrored-self misidentification and hence the unique opportunity to investigate selective cognitive influences in a controlled manner.

### RESPONSES TO DIFFERENT VISUAL MEDIA

Given the apparent success of the hypnotic modeling paradigm so far, the current experiment aimed to better define some of the parameters of hypnotic mirrored-self misidentification. In particular, we focused on the impaired face processing (Factor 1) thought to be responsible for the delusion’s content and sought to extend previous research in three ways. First, we examined whether hypnotic disruptions to self-face recognition generalized to include other visual media. This is directly relevant to the clinical disorder. Some patients with mirrored-self misidentification, for example, remain able to recognize themselves in photographs (Phillips et al., 1996; Breen et al., 2001) and small, handheld mirrors (Kumakura, 1982; Feinberg, 2001). Other patients, however, fail to recognize themselves in photographs (Biringer et al., 1991) or in any type of mirror or reflective surface (Gluckman, 1968; Spangenberg et al., 1998). In healthy participants, there is also evidence that self-face recognition in photographs involves different neural mechanisms to mirror images (Butler et al., 2012; Suddendorf and Butler, 2013).

Second, we examined whether the hypnotic mediated disruptions to face processing affected recognition of *other* people’s faces. In the clinical condition, patients with mirrored-self misidentification vary to the extent that they can recognize images of other people. Whereas some patients recognize people other than themselves in the mirror (e.g., Spangenberg et al., 1998; Breen et al., 2001; Van den Stock et al., 2012), other patients report that all people in the mirror are strangers (Phillips et al., 1996; Breen et al., 2001). Some patients are also impaired in recognizing famous faces (Breen et al., 2001). The current experiment therefore examined whether participants recognized the hypnotist in the mirror, a photograph of person familiar to them (their lecturer), and a series of famous faces.

Finally, we attempted to create a more general deficit in face processing and examined whether the type of impairment specified in the hypnotic suggestion affected participants’ responses. This is theoretically important because there are different views on the type of face processing deficit responsible for mirrored-self misidentification. The account by Phillips et al. (1996) implies that a deficit specific to self-face recognition is responsible for the content of the delusion and explains why some patients can recognize other people in the mirror but not themselves. An alternative account by Breen et al. (2001; see also Langdon, 2011), however, suggests that a more general face processing deficit is responsible for the content of the delusion and is evident in neuropsychological tests of face processing in some patients. Against this background, this experiment compared two suggestions to help disambiguate different types of face processing deficit. The first suggestion was the Factor 1 suggestion for impaired face processing used in previous work (Connors et al., 2012a, 2013, in press-a). This suggestion indirectly implied that participants would only fail to recognize their own face in the mirror, so is referred to here as the *suggestion for impaired self-face recognition.* The second suggestion was a new suggestion designed to impair recognition of all faces. It is referred to here as the *suggestion for impaired general-face recognition*.

### OVERVIEW OF THE CURRENT EXPERIMENT

A hypnotist provided high and low hypnotisable participants with a hypnotic induction and either a suggestion for impaired self-face recognition or a suggestion for impaired general-face recognition. The experimenter then asked participants to identify who they saw in a mirror and in a series of photographs that included participants’ own photograph and a photograph of a high profile lecturer from their psychology course. The experimenter then tested participants’ ability to recognize famous faces in a forced-choice familiarity test (see Young and De Haan, 1988; Rivolta et al., 2010, 2012). After this, the experimenter tested whether participants could identify themselves in a live video image and then a handheld mirror. Next, to assess participants’ understanding of mirrors, the experimenter asked them to define mirrors and to touch a ball that was only visible by its reflection in the mirror on the wall (see Connors and Coltheart, 2011). Finally, the experimenter tested participants’ ability to recognize the hypnotist in the mirror when the hypnotist stood next to them.

This order of tests was not counterbalanced as previous work suggested that some challenges were more likely to break down the delusion than others (Barnier et al., 2011; Connors et al., 2012a). As a result, the tests were presented in a fixed order, starting with those considered to be least confronting and ending with the most confronting. It was expected that both suggestions would generate mirrored-self misidentification in highs, but not lows. In particular, it was expected that whereas highs given the suggestion for impaired self-face recognition would be able to recognize themselves in the other visual media and recognize other faces, highs given the suggestion for impaired general-face recognition would not recognize themselves or other faces in any media.

## MATERIALS AND METHODS

### PARTICIPANTS AND DESIGN

Participants were selected from a pool of 439 students (101 males, 318 females, 20 not disclosed) of mean age 22.06 years (SD = 6.25) on the basis of a 10-item modified version of the *Harvard Group Scale of Hypnotic Susceptibility, Form A* (HGSHS:A; Shor and Orne, 1962). High scorers (participants who scored 7 or greater) and low scorers (participants who scored 3 or less) were invited to participate in the current experiment, which also included an 11-item modified version of the *Stanford Hypnotic Susceptibility Scale, Form C* (SHSS:C; Weitzenhoffer and Hilgard, 1962)^1^ in the same session. Participants received payment ($20 for 1.5 h) for their involvement. A total of 51 participants (16 males, 35 females) of mean age 21.92 years (SD = 6.10) completed this session. Only participants who scored in the range 7–11 (*highs*) or 0–3 (*lows*) on both the HGSHS:A and SHSS:C were included in the analyses.

The final sample consisted of 22 highs (8 males, 14 females) of mean age 21.32 years (SD = 3.85), and 20 lows (7 males, 13 females) of mean age 21.15 years (SD = 5.28). Highs had a mean score of 8.05 (SD = 0.90) on the HGSHS:A and 8.91 (SD = 1.23) on the SHSS:C. Lows had a mean score of 1.60 (SD = 1.19) on the HGSHS:A and 1.55 (SD = 1.19) on the SHSS:C. Participants were tested in a 2 (hypnotisability: high vs. low) × 2 (suggestion: impaired self-face recognition vs. impaired general-face recognition) between-subjects design. Participants were asked not to participate if they had any ongoing psychological condition, problems with substance abuse, or if they had ever suffered a serious head injury or neurological illness. All participants provided written informed consent. Research was approved by the Macquarie University Human Research Ethics Committee.

### MATERIALS AND PROCEDURE

The hypnotist tested participants individually in a 90 mins session. This session consisted of an experimental session and a postexperimental inquiry. Both the experimental session and the postexperimental inquiry were recorded using a video camera.

#### Experimental session

Before the experiment, the hypnotist briefly explained the experiment and obtained participants’ informed consent. Next, the hypnotist took participants’ photograph using a digital camera. The hypnotist then printed the photograph, unbeknownst to participants, who were occupied completing payment forms. To do this, the hypnotist used a Canon Selphy CP780 compact photo printer to produce a standard 14.8 cm × 10.0 cm color photograph. Once printed, the photograph was placed in a photo album containing nine other photographs of faces that were produced using the same camera and printer.

The hypnotist then administered a standard hypnotic induction (~10 min, from the SHSS:C; Weitzenhoffer and Hilgard, 1962). The hypnotist administered the first 10 items from the SHSS:C and scored participants’ responses.

**FIGURE 1:**
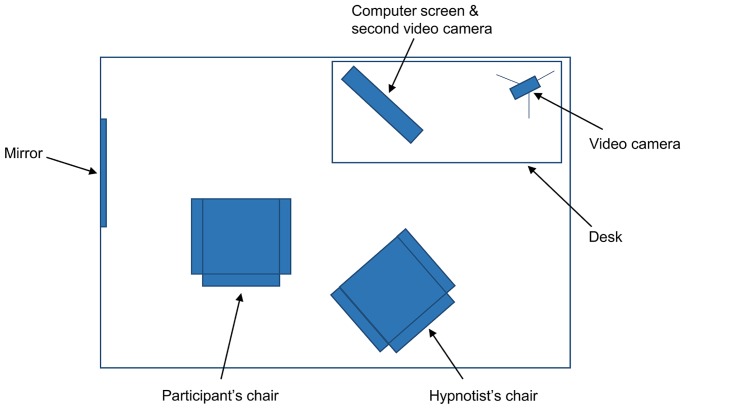
**Topographical view of materials in the experiment.** The mirror was covered with a screen when the participant entered the room. The computer was positioned at 45° from the participant’s chair, though the participant could rotate their chair to face the screen directly.

***Suggestion.*** After these items, the hypnotist uncovered a mirror (~40 cm × 50 cm) that was mounted on a wall next to the participants’ chair. The mirror was positioned so that participants could look directly into it by turning their head to the left and leaning slightly forward (see **Figure 1**). The hypnotist gave participants one of two suggestions for a deficit in face processing. Participants were randomly assigned to receive either the suggestion for impaired self-face recognition (11 highs, 10 lows) or the suggestion for impaired general-face recognition (11 highs, 10 lows). The suggestion for impaired self-face recognition was: 

When you look to your left, there will be a mirror there, and you will see a person in it. When you see this person in the mirror, you will not be able to recognize this person. When you open your eyes and turn your head to your left, whilst remaining as deeply relaxed and comfortably hypnotized as you feel now, you will see a face in the mirror that you will not be able to identify, as if you have never seen this face before.

The suggestion for impaired general-face recognition was:

When you look to your left, there will be a mirror there, and you will see a person in it. When you see this person in the mirror, you will not be able to recognize this person. In fact, when you open your eyes and look around, you will not be able to recognize any person you see. That’s right, whenever you see a face, it will seem unfamiliar to you and you will not be able to recognize who it is. When you open your eyes whilst remaining as deeply relaxed and comfortably hypnotized as you feel now, all faces will seem unfamiliar to you and you will not be able to recognize them.

The hypnotist checked that participants understood the suggestion. The hypnotist then asked participants to slowly open their eyes, turn their head to the left, and look into the mirror.

***Test 1: mirror 1.*** The hypnotist asked participants to identify who they saw in the mirror and to briefly describe them. If participants reported seeing someone other than themselves, the hypnotist asked participants if they had ever seen this person before.

***Test 2: photograph.*** The hypnotist handed participants a photo album that contained the participants’ photograph and nine other photos (eight of unfamiliar faces, one of their lecturer’s face) in one of four fixed randomized orders. The hypnotist asked participants to look at each photo one at a time and to indicate whether the face was familiar or unfamiliar. If participants reported that a face was familiar, the hypnotist asked participants who the person was. When this was completed, the hypnotist took the photo album from participants and asked participants to close their eyes.

***Test 3: Famous faces.*** The hypnotist placed a keyboard on participants’ lap and started the forced choice familiarity task of famous faces on the computer (see Rivolta et al., 2012, for more detail). As shown in **Figure 1**, the computer was positioned in the room approximately 45° to the participants’ right; participants were asked to swivel their chair to face the screen directly. The hypnotist explained to participants that two faces would appear on the computer screen at the same time. One face would belong to someone famous; the other face would belong to someone who was not famous. Participants had to indicate using the keyboard which face was the famous face – that is, whether they thought the famous face was on the left or on the right. The task had 30 trials and involved 30 sets of faces: 30 famous faces (actors, politicians, and musicians who were well known to Australian participants) and 30 unfamiliar faces matched as closely as possible for age, sex, and attractiveness. The famous faces included Jennifer Aniston, Tony Blair, Sandra Bullock, George Bush, Nicholas Cage, Prince Charles, Bill Clinton, George Clooney, Kevin Costner, Tom Cruise, Robert De Niro, Johnny Depp, Cameron Diaz, Leonardo DiCaprio, Clint Eastwood, Queen Elizabeth II, Mel Gibson, Hugh Grant, Tom Hanks, Paris Hilton, Dustin Hoffman, John Howard, Nicole Kidman, Madonna, Kylie Minogue, Brad Pitt, Julia Roberts, John Travolta, Robin Williams, and Catherine Zeta-Jones. The faces were presented as black and white photographs, approximately 10 cm high, on a 51 cm × 32 cm (24″) Macintosh computer screen. The order and positioning (left vs. right) of the famous faces were randomized. Participants were approximately 50 cm from the computer screen and gave their responses by pressing relevant keys on the keyboard. There was no time limit on responses; once a response was selected, the next set of faces appeared. The hypnotist told participants they should try to be as accurate as they could and that if they were unsure they should guess (there was no emphasis on speed). After these instructions, the hypnotist asked participants to open their eyes and begin the task. When the task was completed, the hypnotist took the keyboard from participants.

***Test 4: mirror 2.*** The hypnotist asked participants to look again at the mirror on their left and to identify who they saw. This was done to see whether participants maintained their delusion after the famous faces task.

***Test 5: video.*** The hypnotist activated a live video feed of the participants’ face and shoulders on the computer screen. This required a second video camera, focused on participants, which was concealed above the computer screen. The hypnotist asked participants to look at the computer screen and identify who they saw. The hypnotist then turned off the computer screen.

***Test 6: handheld mirror.*** The hypnotist gave participants a handheld mirror to hold and asked them to identify who they saw in it. The hypnotist then took the handheld mirror from participants.

***Test 7: mirror agnosia.*** The hypnotist first asked participants to define what mirrors are. The hypnotist then held a plastic ball, slightly larger than a tennis ball, above participants’ shoulder and asked them to touch the ball. The hypnotist looked to see whether participants reached towards the ball or towards the ball’s reflection in the mirror (as in mirror agnosia; see Connors and Coltheart, 2011).

***Test 8: mirror 3 and hypnotist’s reflection.*** The hypnotist asked participants to once again look at the mirror on the wall to their left. The hypnotist then moved position so that participants could see the hypnotist’s reflection in the mirror. The hypnotist asked participants who they saw. If participants reported seeing the hypnotist but not themselves, the hypnotist asked them to explain how they could see the hypnotist but not themselves. The hypnotist then touched participants on the shoulder while they were looking in the mirror and asked participants what happened.

***Cancellation and deinduction.*** The hypnotist canceled the suggestion by telling participants that everything was back to normal and that they were able to recognize themselves and other faces, just as they always had been able to. The hypnotist asked participants to look in the mirror once more and checked that they could recognize themselves. Next, the hypnotist gave participants the final SHSS:C suggestion for posthypnotic amnesia and administered the SHSS:C deinduction, which involved gradually awakening participants as the hypnotist counted from 20 to 1. The hypnotist then tested and canceled participants’ posthypnotic amnesia.

#### Postexperimental inquiry

For all media (mirror, photograph, video, handheld mirror), the hypnotist asked participants to describe their experience of looking at it and to rate the extent to which they believed that they were looking at a stranger (1 = *not at all*, 7 = *completely*). The hypnotist also asked participants to repeat the famous faces task to assess whether participants showed different responses when not affected by hypnosis or suggestion. Finally, the hypnotist debriefed participants and thanked them for their time.

#### Coding of responses

After testing all participants, the hypnotist and a rater (who was unaware of the aims of the experiment and the conditions in which participants were tested) independently examined the videotape records of the experiment. The two raters scored whether or not participants recognized themselves in each of the different visual media. The raters also scored whether or not participants recognized their lecturer in a photograph and the hypnotist in the mirror. Interrater reliability was 100%.

## RESULTS

### EXPERIENCING THE DELUSION

Participants were scored as passing the suggestion if they identified their reflection in the mirror as someone other than themselves. Overall, 9 (82%) highs given the suggestion for impaired self-face recognition and 5 (46%) highs given the suggestion for impaired general-face recognition passed the suggestion. Fisher’s exact test showed that this difference did not reach statistical significance, *p* = 0.18. No lows passed the suggestion. The 14 highs who reported seeing a stranger were asked if they had ever seen this person before. Of these, 10 (71%; 8 impaired self-face recognition, 2 impaired general-face recognition) said they had never seen the person before, 2 (14%; 2 impaired general-face recognition) said they had seen the person before, and 2 (14%; 2 impaired self-face recognition) were unsure. Consistent with previous research (Connors et al., 2013, 2014), a *post hoc* analysis revealed that highs who passed the suggestion had higher SHSS:C scores than highs who failed the suggestion, *F*(1,18) = 4.56, *p* = 0.05, $\eta_{p}^{2}$ = 0.20, but did not differ on HGSHS:A scores, *F*(1,18) = 0.24, *p* = 0.63, $\eta_{p}^{2}$ = 0.01. The remainder of the results focus on the highs who passed the suggestion unless otherwise specified.

### RESPONSE TO THE DIFFERENT MEDIA

The responses of participants to the different visual media are shown in **Table 1**. Participants were scored as being impaired on these tests if they failed to identify themselves. Statistical comparisons using Fisher’s exact test revealed that more highs given the impaired self-face recognition suggestion failed to recognize themselves in the photograph (*p* = 0.02) and in the mirror the second time it was presented (*p* = 0.02) than highs who received the impaired general-face recognition suggestion. There was, however, no differences between suggestions in terms of highs’ responses to the video (*p* = 0.15), handheld mirror (*p* = 0.59), or the mirror on its third presentation (*p* = 1.00).

**Table 1:** The number and percentage of participants who failed the visual tests.

	Highs		Lows	
	Impaired self-face *(n = 11)*	Impaired general-face *(n = 11)*	Impaired self-face *(n = 10)*	Impaired general-face *(n = 10)*
1. Mirror 1	9 (82%)	5 (45%)	0 (0%)	0 (0%)
2. Photograph	7 (64%)	1 (9%)	0 (0%)	0 (0%)
*Lecturer’s photograph*	3 (27%)	5 (45%)	2 (20%)	2 (20%)
3. *Famous Faces*	0 (0%)	2 (18%)	0 (0%)	0 (0%)
4. Mirror 2	7 (64%)	1 (9%)	0 (0%)	0 (0%)
5. Video	5 (45%)	1 (9%)	0 (0%)	0 (0%)
6. Handheld mirror	3 (27%)	1 (9%)	0 (0%)	0 (0%)
7. Mirror agnosia*	0 (0%)	0 (0%)	0 (0%)	0 (0%)
8. Mirror 3	3 (27%)	1 (9%)	0 (0%)	0 (0%)
*Hypnotist in mirror*	0 (0%)	1 (9%)	0 (0%)	0 (0%)

Tests in italics involved recognition of other people; *test does not involve self or other recognition.

Overall, three highs (27%) given the impaired self-face recognition suggestion and one high (9%) given the impaired general-face recognition suggestion failed to recognize themselves in all visual media – these four highs maintained the suggested experience across all tests. In contrast, two highs (18%) given the impaired self-face recognition suggestion and six highs (55%) given the impaired general-face recognition suggestion recognized themselves in all visual media – these eight highs failed the suggested experience. The remaining six highs (55%) given the impaired self-face recognition suggestion and four highs (36%) given the impaired general-face recognition suggestion showed mixed responses – these ten highs recognized themselves in some media but not others. Some of these highs initially failed to recognize themselves in the mirror but breached the suggested experience during the course of the experiment. In the case of the impaired self-face recognition suggestion, two of the nine highs (22%) who initially passed the suggestion reported recognizing themselves in the mirror the second time it was presented. In the case of the impaired general-face recognition suggestion, four of the five highs (80%) who initially passed the suggestion reported recognizing themselves in the mirror the second time it was presented. This left only one high given the impaired general-face recognition suggestion who failed to recognize themselves across different visual media. These findings implied that the experience of the impaired general-face recognition suggestion broke down more quickly than the experience of the impaired self-face recognition suggestion.

Despite this, highs given the impaired general-face recognition suggestion were more likely to not recognize other people than highs given the impaired self-face recognition suggestion. A greater proportion of highs who passed the impaired general-face recognition suggestion failed to recognize their lecturer’s photograph or the hypnotist in the mirror than highs who passed the impaired self-face suggestion (**Table 1**). This difference between suggestions was also evident in the famous faces task. Participants were scored as being impaired on the famous faces task if their scores were at chance during the experiment, but significantly above it once the suggestion was canceled. As shown in **Table 1**, two highs (18%) given the suggestion for impaired general-face recognition met this criterion: They scored 10/30 and 14/30 during the experiment, but were unimpaired when they repeated the task in the postexperimental inquiry and scored 30/30 and 28/30, respectively. In contrast, no highs given the impaired self-face suggestion and no lows had difficulty completing the famous faces task (for highs, *M* = 26.68, SD = 5.08; for lows, *M* = 27.35, SD = 2.23). A repeated-measures ANOVA, however, revealed no group differences between highs and lows or between the two suggestions, most likely due to the small number of participants experiencing these effects (all *F*s < 3.22, all *p*s > 0.08, all $\eta_{p}^{2}$s < 0.08).

All participants’ ratings of belief in the postexperimental inquiry are shown in **Table 2**. Ratings across the different media were compared using a mixed ANOVA with between-subject factors of hypnotisability (high vs. low) and suggestion (impaired self-face recognition vs. impaired general-face recognition) and a within-subject factor of visual media (mirror, photograph, video, handheld mirror). In all media, highs rated their belief that they were looking at a stranger higher than lows, *F*(1,38) = 33.77, *p* < 0.01, $\eta_{p}^{2}$ = 0.47. There was also a significant difference between visual media, *F*(3,38) = 11.25, *p* < 0.01, $\eta_{p}^{2}$ = 0.23, and a significant interaction between hypnotisability and visual media, *F*(3,38) = 8.60, *p* < 0.01, $\eta_{p}^{2}$ = 0.19. Whereas highs overall reported moderate ratings for the mirror and gave declining ratings thereafter, lows reported consistently low ratings for all visual media. There was no difference between suggestions and no interactions between hypnotisability and suggestion (all *F*s < 3.63, all *p*s > 0.07, all $\eta_{p}^{2}$s < 0.09). Overall, this indicates that the effects were limited to highs, effects declined somewhat over the visual media, and there were no clear differences between the two suggestions.

**Table 2:** The postexperimental ratings of all participants regarding the extent to which they believed they were looking at a stranger in each of the visual media.

	Highs		Lows	
	Impaired self-face	Impaired general-face	Impaired self-face	Impaired general-face
Mirror	4.73 (1.49)	3.45 (2.02)	1.10 (0.32)	1.10 (0.32)
Photograph	3.73 (1.85)	2.55 (1.86)	1.10 (0.32)	1.70 (0.95)
Video	3.27 (1.49)	2.00 (1.84)	1.00 (0.00)	1.10 (0.32)
Handheld Mirror	2.36 (1.43)	1.91 (1.76)	1.00 (0.00)	1.00 (0.00)

*Ratings were made on a scale of 1-7 (1 = *not at all*, 7 = *completely*). Standard deviations are in parentheses.*

During the postexperimental inquiry, highs who passed the suggestion described a compelling experience. When asked about their experience of looking in the mirror, highs given the suggestion for impaired self-face recognition made comments like, “It just wasn’t me. I thought that if I looked in the mirror, I would see me, but it didn’t look or feel like me.” Another high given this suggestion said, “It was a bit bewildering actually … I was looking at someone in there but I couldn’t register who it was. I was confused. I thought, ‘Who is this person?”’ Highs given the suggestion for impaired general-face recognition reported similar experiences. One high given this suggestion, for example, said, “It was weird. I know when you look in the mirror, it’s meant to be you, but it was just unfamiliar. I just didn’t recognize it was me.” Another high given this suggestion said, “I actually felt like there was actually another person in the mirror. That another person was looking back at me. They felt familiar, but I didn’t know who they were.”

When asked about the other visual media, many highs reported similar experiences as when looking in the mirror. When describing the experience of looking at his photo, one high given the suggestion for impaired self-face recognition said, “I remember looking at it and being confused, like I was in the mirror. I felt as if I should know who it was, but I didn’t.” Another high given this suggestion described looking at her photo in a similar way: “I eventually came to the conclusion that I had never seen this person before. It was a similar experience to when I was looking in the mirror.” When asked about the live video, highs said, “It felt weird, very similar to the feeling I had when I looked in the mirror. It just felt like I should be seeing me but it wasn’t me. Sort of familiar, like feeling familiar with it, but also very unfamiliar.” Other highs made comments like, “He looked very familiar. It looked like the guy in the mirror” and “I didn’t think it looked like me. It just felt like someone really foreign, someone I wasn’t familiar with.”

When asked about the handheld mirror during the postexperimental inquiry, one high said he saw, “The same thing [as the mirror]. Just familiar but unfamiliar. Not what I would normally expect to see and feel.” The one high who received the suggestion for impaired general-face recognition and maintained the delusion reported that she did not remember her experiences looking in the mirror. Such unsuggested posthypnotic amnesia is rare (Hilgard and Cooper, 1965; Hilgard, 1966; Cooper, 1979), but was also present in a participant in a previous experiment (Connors et al., 2012b). The other high given this suggestion who was impaired on the famous faces task described his experience as very compelling: “I found it extremely difficult. They both just looked famous, I could not really tell. Sometimes I could tell them apart after a while but sometimes I just had no clue who it was.” However, these highs were in the minority; the majority of highs reported recognizing the famous faces and recognizing themselves in the handheld mirror.

A number of highs who did not show the delusion reported that they had some difficulty recognizing themselves. Three highs who received the suggestion for impaired general-face recognition said that they were initially unsure who they were looking at. Two of these highs said that they concluded it was them when they noticed the person in the mirror was wearing the same clothes as them, and the third said he recognized it was him when he saw the person move at the same time as he did. Likewise, four highs who displayed the delusion and failed to recognize themselves in the mirror (two impaired self-face recognition, two impaired general-face recognition) reported some initial difficulty recognizing themselves in the live video. These highs said they concluded it was themselves because they recognized the room they were in. A further three highs who experienced the delusion after the suggestion for impaired self-face recognition said that they had difficulty recognizing themselves in the handheld mirror but that the fact that they were holding and controlling it led them to believe it was themselves. Finally, one high (given the suggestion for impaired self-face recognition) breached her delusion after the hypnotist appeared next to her. This participant described having difficulty reconciling her subjective experience with what she knew to be true: “When you moved behind me, I realized it had to be me in the mirror. I still had some doubts though. My experience was that I still didn’t think it was me, but logically it had to be me.”

## DISCUSSION

### OVERVIEW

Both hypnotic suggestions disrupted the ability of highs to recognize themselves in the mirror. Highs, however, showed a different pattern of responses to the other visual media depending on the nature of the suggestion received. When tested on their ability to recognize themselves in other visual media, a proportion of highs given the suggestion for impaired self-face recognition failed also to recognize themselves in a photograph, in a live video, and in a handheld mirror. In contrast, only one high who received the suggestion for impaired general-face recognition failed to recognize herself in other visual media. When tested on their ability to recognize other faces using the famous faces task, no highs given the suggestion for impaired self-face recognition were impaired, whereas two highs given the suggestion for impaired general-face recognition were impaired. Although these findings are obviously limited by the small numbers of highs passing and maintaining the delusion, the findings show the potential for these two suggestions to model different aspects of mirrored-self misidentification.

### SELF-FACE RECOGNITION IN DIFFERENT VISUAL MEDIA

As in previous work (Connors et al., 2012a), a hypnotic suggestion for impaired self-face recognition was able to recreate the surface features of the mirrored-self misidentification delusion. In particular, participants reported that their reflection was not themselves and maintained this belief over time. The current experiment extended previous findings by examining how participants with the hypnotic delusion responded to different visual media. The findings show that this suggestion affected the ability of some highs to recognize themselves in other visual media, despite not directly specifying this in the suggestion. As expected, however, the suggestion for impaired self-face recognition did not impair the ability of highs to recognize other people. Highs given this suggestion correctly identified their lecturer’s photograph, identified the hypnotist in the mirror, and were not impaired in the famous faces task. These highs also showed an intact procedural understanding of mirrors. These findings indicate hypnotic suggestion might be able to selectively impair self-face recognition in some participants. Nevertheless, this pattern of responses differs from some clinical patients with mirrored-self misidentification who often show more general deficits in face processing (Phillips et al., 1996; Breen et al., 2001; Van den Stock et al., 2012).

This experiment used a new suggestion – a suggestion for impaired general-face recognition. This suggestion for impaired general-face recognition, however, did not seem to be as successful at generating mirrored-self misidentification as the original suggestion. Fewer participants receiving this suggestion reported the delusion than those receiving the original suggestion, although this difference did not reach statistical significance. The resulting delusion also broke down quickly, leaving only one participant who maintained the delusion through all the tests. This participant failed to recognize herself or other people in any of the different visual tests, yet showed an intact procedural understanding of mirrors. Although limited by the single participant, this high demonstrates that it is possible to generate a general face processing deficit using hypnotic suggestion. Importantly, two highs given the suggestion for impaired general-face processing were impaired on the famous faces task. These participants performed at a level very similar to patients with prosopagnosia, a condition in which participants have difficulty recognizing faces (Behrmann and Avidan, 2005; Rivolta et al., 2013) and show impairments in recognizing famous faces in forced choice tasks (Young and De Haan, 1988; Rivolta et al., 2012). Unlike patients, however, these two participants showed no sign of impairment once the suggestion was canceled. These findings indicate that hypnotic suggestion can create a general face processing deficit that can be measured on a formal neuropsychological test. The findings are consistent with Oakley and Halligan (2013), who used hypnotic suggestion to model prosopagnosia in a single participant. The current experiment replicated these finding using a more stringent, forced-choice measure, though only in two participants. Together, these findings indicate that hypnotic suggestion may be able to disrupt face processing in certain high hypnotisable participants. However, the fact that only 18% of highs given this suggestion showed this deficit reveals the difficulty of this type of hypnotic suggestion (as a comparison, 23% of highs in this experiment passed the suggestion for negative visual hallucination – to not see a specific object – in the SHSS:C; this suggestion is known to be difficult even for highs; Hilgard, 1965).

Other factors may have also prevented some participants from responding to the suggestion for impaired general-face recognition. Three participants reported in the postexperimental inquiry that they felt anxious when they heard this suggestion and were worried about what it would be like to not recognize faces. None of these participants experienced the delusion and it is possible that their anxiety interfered with their response to the suggestion. A fourth participant reported in the postexperimental inquiry that she had difficulty imagining what it would be like to not recognize faces. This participant likewise did not develop the delusion and it is possible that her difficulty anticipating the effects of the suggestion prevented her from responding. Overall, these findings highlight a limitation of using hypnosis to model clinical conditions. Responses are affected by factors such as the participants’ expectations and interpretations, as well as the relative difficulty of the suggestion. It is thus not the verbatim suggestion, but the participants’ interpretation of the suggestion and ability to experience it that shapes their response (McConkey, 1991, 2008). It is important to consider these factors when designing a hypnotic analog (see Connors et al., 2012b).

For both suggestions, a proportion of highs breached the hypnotic delusion during the visual tests. The visual tests, although not designed to challenge participants’ hypnotic experiences, provided accumulating evidence against the hypnotic delusion and this may have led some highs to breach their delusion. As a result, it is difficult to compare the different tests because they were given in a single order that was designed to minimize breaching. However, the fact that some highs breached the delusion is consistent with previous research, which found that directly challenging the hypnotic delusion with confronting evidence led some participants to breach the delusion and report seeing themselves in the mirror (Connors et al., 2012a). The finding is also consistent with research that has found that a proportion of highs experiencing a hypnotic delusion (Noble and McConkey, 1995; Cox and Barnier, 2009) or posthypnotic amnesia (Kihlstrom et al., 1980; McConkey and Sheehan, 1981; Coe, 1989; Coe and Sluis, 1989) breach their experience in response to challenges. Hypnotic effects require participants to resolve the conflict between objective reality and the suggested experience (McConkey, 1983; Mallard and Bryant, 2006). Challenges both draw attention to and increase this conflict, leading some participants to breach the suggested effect. Nevertheless, a proportion of highs maintain their hypnotic responses in the face of confronting evidence and an important question for future research is whether particular individual differences predict whether participants maintain or breach their hypnotic experience (Connors et al., 2014).

### HETEROGENEITY IN RESPONSES

As in previous work (Connors et al., 2012a), hypnotized participants displayed considerable variation in their responses to hypnotic suggestions and this variation corresponds to heterogeneity seen in clinical reports. Both hypnotized participants and clinical patients, for example, vary in the extent to which they recognized themselves in photographs, video, and handheld mirrors (Biringer et al., 1991; Breen et al., 2001; Connors and Coltheart, 2011). For both hypnotized participants and clinical patients, it is likely that the specific properties of the different visual media influence self-recognition. These properties may, in part, explain why some participants (and patients) recognize themselves in some visual media but not in others. Mirrors, for example, offer movement and depth cues that are not present in photographs. As a result, mirrors provide a highly realistic image that could be confused with a real person, whereas photographs provide a static, two-dimensional image that is unlikely to be confused in the same way (see also Butler et al., 2012; Suddendorf and Butler, 2013). In a similar way, a handheld mirror shows just the face in its narrow field of vision and is accompanied with greater physical control of the visual image than a larger mirror on the wall. All these cues could lead some participants and patients to identify themselves in a handheld mirror, despite being unable to identify themselves in a larger mirror on the wall and a clear understanding of how mirrors operate.

A large part of the variability, however, may also originate from the participants and patients themselves. Within the hypnotic model, for example, there are a number of sources of variation. Highs might interpret the same verbal suggestion in different ways to each other (see McConkey, 1991, 2008) and/or differ in their ability to experience specific types of hypnotic effects (see Woody et al., 2005). As a result, they may have different responses to the visual media. Highs also could use different cognitive strategies to experience the suggestion and this could lead to different responses (McConkey, 1991, 2008; McConkey and Barnier, 2004). Previous research, for example, has shown that highs using a constructive strategy (in which they actively use cognitive strategies to experience the hypnotic suggestion) were more likely to pass a suggestion for hypnotic blindness than participants using a concentrative strategy (in which they focused on the hypnotist’s words; Bryant and McConkey, 1990). In addition, highs could vary in terms of how completely they respond to the suggestion (see Spanos, 1986). Cognitive-delusory suggestions tend to be more difficult to experience, even for highs, and highs could vary in their ability to generate a compelling and vivid experience.

In the clinical delusion, there are a number of other sources of variation. The variability, for example, could be due to the specific aspects of face processing that are impaired (see Langdon, 2011). The influential model of face processing by Bruce and Young (1986) holds that face processing involves a sequence of stages. These stages include encoding the structural properties of a face, experiencing a sense of familiarity if the face is known, accessing semantic information about the person, and naming the person. Patients who only have impairment at a late stage of face processing (such as in accessing semantic information or naming) may still experience a sense of familiarity when looking at images of themselves in some media. This sense of familiarity could provide the basis of self-face recognition in these instances (see Mandler, 1980). In contrast, patients who have more pervasive impairments or impairment at an earlier stage of face processing (such as in encoding the structural properties of faces) may fail to experience even this sense of familiarity when looking at images of themselves. As a result, these patients may fail to recognize themselves in all media. Future research could investigate this possibility by directly testing clinical patients and potentially also by using hypnotic models.

### IMPLICATIONS AND FUTURE DIRECTIONS

The current study has a number of limitations that could be addressed in future work. Given the significant variability evident among participants, larger sample sizes will be required to fully define the nature of the face-perception deficits and examine the role of individual differences. Future research could also formally test for both familiarity and recognition, use other types of face processing tests, and use larger numbers of trials to detect smaller effects. In addition, future research could examine the specific visual cues that participants use to recognize themselves in different media. Research, for example, could vary the size of the image in each media, use time delayed video footage to remove contingency cues, and disguise the video monitor as a mirror by placing a frame around it to alter expectations associated with the medium. As mirrors present images in a different orientation to photographs, reversing the axis of left and right, future research could also examine the role of this visual transformation by presenting photographs of participants and famous faces in this orientation. Finally, given that several participants suggested that their anxiety might have prevented them from experiencing the impaired general-face recognition suggestion, future research could consider revising the wording of this particular suggestion to make it appear more benign. This could be done, for example, by emphasizing that the effect would only be temporary and by suggesting that participants might find the experience both pleasant and interesting.

In addition to these issues, we acknowledge a number of important differences between clinical delusions and hypnotic models. Clinical delusions are functionally disruptive, and typically endure for long periods of time and across different contexts. In contrast, hypnotic delusions are short-lived, highly contextualized, and limited to the laboratory (see Barnier et al., 2008; Cox and Barnier, 2010). These differences between clinical and hypnotically suggested delusions obviously limit the ability to generalize experimental findings to clinical patients. For example, the longer duration of clinical delusions may lead to more extensive elaboration of the delusion, compared to the shorter exposure in otherwise healthy controls and where delusions are observed at their inception. Indeed, clinical patients with mirrored-self misidentification can often seem accustomed, even indifferent, to the stranger and attribute names and details to them (Breen et al., 2001). In contrast, many hypnotized participants appear surprised or shocked to see a stranger in the mirror and report not having seen the person before or knowing who they are. This difference in timeframe may be useful to simulate the experiences of patients when their delusion first forms, which is usually not possible to study directly in clinical patients. It is also important to note, though, that some aspects of the delusion may not be captured in the hypnotic model as they may require the persistence of the experience over long periods of time and across different contexts. It is also important to recognize that, despite our focus on a monothematic delusion, many patients reporting this belief may experience other clinically related symptoms as a result of their overall condition (see Brodaty et al., 2013a,b).

Despite these differences, specific hypnotic suggestions could be used to test theoretical accounts of other clinical delusions. Other delusions, such as Capgras (the belief that a loved one is replaced by a visually similar impostor) and Frégoli (the belief that familiar people are following one around in disguise), may be due, in part, to disorders in face processing (Ellis and Young, 1990; see also Coltheart et al., 2011; Langdon, 2011). In Capgras delusion, loss of autonomic responsiveness to faces may lead to the idea that a known person has been replaced by an impostor. In Frégoli delusion, heightened autonomic responsiveness to faces may lead to the idea that strangers are known people in disguise. Future research could use hypnotic suggestion to manipulate face processing and model these other clinical delusions. According to Langdon and Coltheart’s (2000) two-factor theory, a deficit in belief evaluation is also necessary for a delusion to form. In other research we have conducted (Connors et al., 2012a, 2013), we have found that a hypnotic induction can model this Factor 2 and specifically disrupt belief evaluation. It remains possible, however, that some individuals may not need to have a deficit in belief evaluation hypnotically induced in order to accept a suggestion for a delusional belief (Connors et al., 2013). In particular, pre-existing differences in the belief evaluation process could themselves act as Factor 2 and predispose certain individuals to delusions. Within hypnosis, there is also some evidence of variability in how highs rate their subjective experiences of a hypnotic induction (Terhune and Cardeña, 2010) and in how they objectively respond to suggestions following different types of hypnotic inductions (Brown et al., 2001). An important direction for future research, therefore, is to characterize the nature of Factor 2 in both clinical patients and hypnotic analog.

Hypnotic suggestions can also be used to investigate face processing independently of delusional belief. Specific suggestions can be designed to selectively impair specific stages of face processing within cognitive models. Adopting Bruce and Young’s (1986) influential account, for example, a suggestion to not be able to discriminate features in faces could disrupt the structural encoding of faces, a suggestion to not recognize familiar faces could disrupt face recognition units that represent previously seen faces, and a suggestion to not be able to recall personal information about faces could disrupt the person identity nodes that link recognized faces to knowledge about the people. The ability to produce these effects on demand makes hypnotic suggestion particularly suited to neuroimaging (Oakley and Halligan, 2009, 2013; Woody and Szechtman, 2011). Future research could examine the underlying functional neuroanatomy and altered functional connectivity associated with hypnotic disruptions to face processing. Such investigations have the potential to inform neural models of face processing (see Gobbini and Haxby, 2007; Haxby and Gobbini, 2011; Kanwisher and Barton, 2011). While it is important to carefully screen participants both on their hypnotisability and their ability to experience these specific suggestions in order to carry out such research, hypnotic suggestion provides a unique means of examining how higher-order cognitive processes influence different stages of face perception. As such, hypnosis offers considerable promise as a methodology to study both face perception and its pathologies.

## Conflict of Interest Statement

The authors declare that the research was conducted in the absence of any commercial or financial relationships that could be construed as a potential conflict of interest.

## Footnotes

1 The 10-item modified HGSHS:A included: head falling, eye closure, hand lowering, finger lock, moving hands together, communication inhibition, experiencing of fly, eye catalepsy, posthypnotic suggestion, and posthypnotic amnesia; arm rigidity and arm immobilization items were removed to ensure that the procedure could be conducted within the time limits of a 1 h class. The 11-item tailored SHSS:C included: hand lowering, moving hands apart, mosquito hallucination, taste hallucination, arm rigidity, dream, age regression, arm immobilization, anosmia, negative visual hallucination, and posthypnotic amnesia; the auditory hallucination item was removed to ensure that the procedure could be conducted within the time limits of a 1 h individual session.
